# Inter-Rater Reliability of the Feline Grimace Scale in Cats Undergoing Dental Extractions

**DOI:** 10.3389/fvets.2020.00302

**Published:** 2020-05-29

**Authors:** Ryota Watanabe, Graeme M. Doodnaught, Marina C. Evangelista, Beatriz P. Monteiro, Hélène L. M. Ruel, Paulo V. Steagall

**Affiliations:** ^1^Department of Clinical Sciences, Faculty of Veterinary Medicine, Université de Montréal, Saint-Hyacinthe, QC, Canada; ^2^Department of Veterinary Clinical Medicine, College of Veterinary Medicine, University of Illinois, Urbana, IL, United States

**Keywords:** feline, dentistry, analgesia, periodontal disease, dental pain, pain assessment, facial expression, Feline Grimace Scale

## Abstract

This study aimed to evaluate the inter-rater reliability of the Feline Grimace Scale (FGS) in cats undergoing dental extractions and the effects of the caregiver's presence on the FGS scores. Twenty-four cats (6 ± 3.3 years old; 4.9 ± 1.7 kg) undergoing oral treatment were included in a prospective, blinded, randomized, clinical study. They underwent treatment under general anesthesia (acepromazine-hydromorphone-propofol-isoflurane-meloxicam-local anesthetic blocks) at day 1 and were discharged at day 6. Images of cat faces were captured from video recordings with or without the caregiver's presence at 6 h postoperatively (day 1), day 6, and before and after rescue analgesia. Images were randomized and independently evaluated by four raters using the FGS [five action units (AU): ear position, orbital tightening, muzzle tension, whiskers change, and head position; score 0–2 for each]. Inter-rater reliability and the effects of the caregiver's presence were analyzed with intraclass correlation coefficient [single measures (95% confidence interval)] and the Wilcoxon signed-rank test, respectively (*p* < 0.05). A total of 91 images were scored. Total FGS scores showed good inter-rater reliability [0.84 (0.77–0.89)]. Reliability for each AU was: ears [0.68 (0.55–0.78)], orbital tightening [0.76 (0.65–0.84)], muzzle [0.56 (0.43–0.69)], whiskers [0.64 (0.50–0.76)], and head position [0.74 (0.63–0.82)]. The FGS scores were not different with [0.075 (0–0.325)] or without [0.088 (0–0.525)] the caregivers' presence (*p* = 0.12). The FGS is a reliable tool for pain assessment in cats undergoing dental extractions. The caregiver's presence did not affect FGS scores.

## Introduction

Oral disease is often observed in veterinary medicine (1). Our laboratory revealed that cats with severe oral disease requiring multiple tooth extractions had specific pain-induced behaviors, higher pain scores, changes in serum inflammatory cytokines, and lower food intake when compared with cats with no/minimal oral disease (2, 3). There are three pain scales with validation for feline pain assessment: Glasgow Composite Measure Pain Scale-Feline (CMPS-F) (4), UNESP-Botucatu multidimensional composite pain scale (5) and the recent Feline Grimace Scale (6, 7). However, these tools have not been used specifically in the context of pain caused by oral disease. The main challenge related to the use of the first two pain scales is that some questions are not applicable to cats with oral pain. For example, cats with oral pain often do not pay attention to the surgical area and it is often difficult to palpate a painful area (i.e., inside the oral cavity), which would be key behaviors in cats with other sources of pain including the abdomen and limbs (2). Thus, oral pain could be underestimated resulting in delays for analgesic intervention.

The Feline Grimace Scale (FGS) has been recently published and it comprises five action units (AU): eyes, ears, muzzle, whiskers, and head position. The instrument was developed and validated for naturally occurring pain of different sources and intensities (6). The clinical applicability of the FGS has been confirmed by comparing image with real-time assessment. In brief, minimal bias and narrow limits of agreement were observed between both methods of assessment (7). However, the FGS has not been specifically tested for assessment of oral pain, yet. In the authors' experience, multiple dental extractions can lead to facial edema which might influence the FGS scores. Therefore, there is an interest to understand the application and reliability of the FGS in cats undergoing oral treatment including dental extractions.

Pain assessment in the clinical setting requires real-time evaluation for early analgesic intervention. In laboratory animals (i.e., mice and rats), it is known that the presence of male evaluators affects pain scores, producing stress-induced pain inhibition (8). It is not known if a similar phenomenon also happens in cats.

The objectives of this study were to evaluate the inter-rater reliability of the FGS after oral treatment and the effect of the caregiver's presence on FGS scores. Our hypotheses were that the scores from different raters would be reliable and the presence of the caregiver would decrease the FGS score.

## Materials and Methods

### Study Design

Data for this study were obtained from a previously reported clinical trial involving dentistry, nutrition, pain management and behavior in cats before and after dental extractions (2, 3). The study was approved by the Institutional Animal Care and Use Committee of the Université de Montréal (protocol 17-Rech-1890) and performed at the Centre hospitalier universitaire vétérinaire (CHUV), Faculty of Veterinary Medicine, Université de Montréal, between July 2017 and February 2018. The study is reported according to the CONSORT guidelines (http://www.consort-statement.org). The study design was a prospective, blinded, randomized clinical trial.

### Animals

Twenty-four healthy cats (6 ± 3.3 years old; 4.9 ± 1.7 kg, 11 and 13 neutered males and females, respectively) with or without naturally occurring oral disease were included. Cats were considered healthy based on history, medical records, physical examination, complete blood count and biochemical panel. Recruitment of cats from shelter facilities was performed by two investigators (PS and BM) after informed written consent. All cats were admitted the day before dental procedures (day 0), and they underwent dental treatment under general anesthesia on day 1 and were discharged on day 6. They were housed in stainless steel cages in a cat-only ward and had free access to water, litter box and bedding. The amount of food offered was calculated based on caloric requirement as previously reported (2).

### Inclusion and Exclusion Criteria

Cats were divided in one of two groups according to the severity of oral disease: no/minimal oral disease (*n* = 12) or severe oral disease requiring dental treatment (*n* = 12) (2). Diagnostic and treatments including dental examination (evaluation of gingival and calculus index, periodontal disease staging, and the number of missing tooth and tooth resorption), radiography, scaling, polishing, and/or extractions were performed as needed. Enrollment into either no/minimal or severe oral disease group in each cat was determined after dental treatment based on the size and number of extracted teeth (2). Cats with a body condition score of <3 or more than seven out of nine were not included. Cats with fearful behaviors, concurrent medical conditions, systemic disease, and the use of analgesics and/or antibiotics within a period up to 10 days before presentation were also not included.

### Anesthesia, Analgesia and Dental Treatment

Detailed description of anesthetic and monitoring procedures is available elsewhere (2). Briefly, premedication included the intramuscular (IM) administration of acepromazine (0.02 mg/kg; 1 mg/mL, Acepromazine maleate, Gentès & Bolduc, Saint-Hyacinthe, QC, Canada) and hydromorphone (0.1 mg/kg; 2 mg/mL, Hydromorphone hydrochloride, Sandoz, Boucherville, QC, Canada). Anesthesia was induced with intravenous (IV) propofol (10 mg/mL, Propoflo 28, Zoetis, Kirkland, QC, Canada) and maintained with isoflurane (Isoflurane USP, Fresenius Kabi, Toronto, ON, Canada) in oxygen. Under general anesthesia, complete dental examination, radiography, scaling/polishing and tooth extractions (if needed) were performed by a board-certified individual and a 3rd-year resident of the American Veterinary Dental College. Cats requiring tooth extraction received local anesthetic blocks with bupivacaine (5 mg/mL, Sensorcaine, AstraZeneca, ON, Canada) using a 1 mL syringe and a 25-G needle (up to a total of 2 mg/kg) as needed including infraorbital, maxillary, and/or inferior alveolar mandibular nerve blocks ~20 min before extractions. At the end of dental treatment, all cats received meloxicam (0.2 mg/kg; Metacam 5 mg/mL Solution for Injection; Boehringer Ingelheim, Burlington, ON, Canada) subcutaneously. Oral administration of meloxicam (0.05 mg/kg, Metacam 0.5 mg/mL Oral Suspension for Cats; Boehringer Ingelheim, Burlington, ON, Canada) was continued at 24, 48, and 72 h after the first dose according to label recommendations in Canada.

### Real-Time Pain Assessment, Video Recording and Video Editing

Real-time pain assessment was performed by one male observer [RW] using the CMPS-F at 23 different time-points from day 0 to 6. This observer was unaware of the oral condition and/or treatment of the cat. Video recordings were performed at 9 different time-points from day 0 to 6 for the study of orofacial pain-related behaviors using a wide-angle lens camera (GoPro Hero 5, GoPro, Riverside, CA, USA) set between the cage bars and remotely controlled by a smartphone (iPhone7, Apple Inc, Cupertino, CA, USA) (3). Cats were moved to a specific cage for video recording that included better lighting. After a 5-min acclimation to the new cage, 10-min videos were recorded for assessment of general (without the observer in the ward), playing, feeding and post-feeding behaviors (with the observer in room) for the purpose of studying different aspects of oral pain-induced pain behaviors (3). Briefly, the recordings of general and playing behavior were aimed to observe behaviors without interaction with the observer and the behaviors during playing with the observer using a ribbon toy, respectively. Data from selected time-points in which both real-time pain assessment and video recording had been performed were used in this study. These included the following four time-points: at 6 h postoperatively on day 1, at 8 am on day 6 and those recorded before and after rescue analgesia. These time points were chosen to represent a wide range of images of painful and non-painful cats. Video editing (trimming) was performed by the same observer [RW] using a video player software (QuickTime Player 10.5, Apple Inc, Cupertino, CA, USA) to obtain videos without the presence of the caregiver during recordings of general behaviors, and videos with the presence of the caregiver during recordings of playing behaviors. For the latter case, only recordings performed when the caregiver had entered the room but before playing with the cat using a ribbon toy were used.

During real-time pain assessment, if a cat had CMPS-F scores ≥ 5/20, rescue analgesia was administered with hydromorphone [0.05 mg/kg IV, if the IV catheter was in place (i.e., first 24 h after surgery) or 0.1 mg/kg IM, if the IV catheter had been removed]. CMPS-F scores were re-assessed 30 min after rescue analgesia. Additional 5-min videos were recorded immediately before rescue analgesia and 30 min after the administration of hydromorphone without the caregiver in the room.

### Image Collection

**Figure 1:**
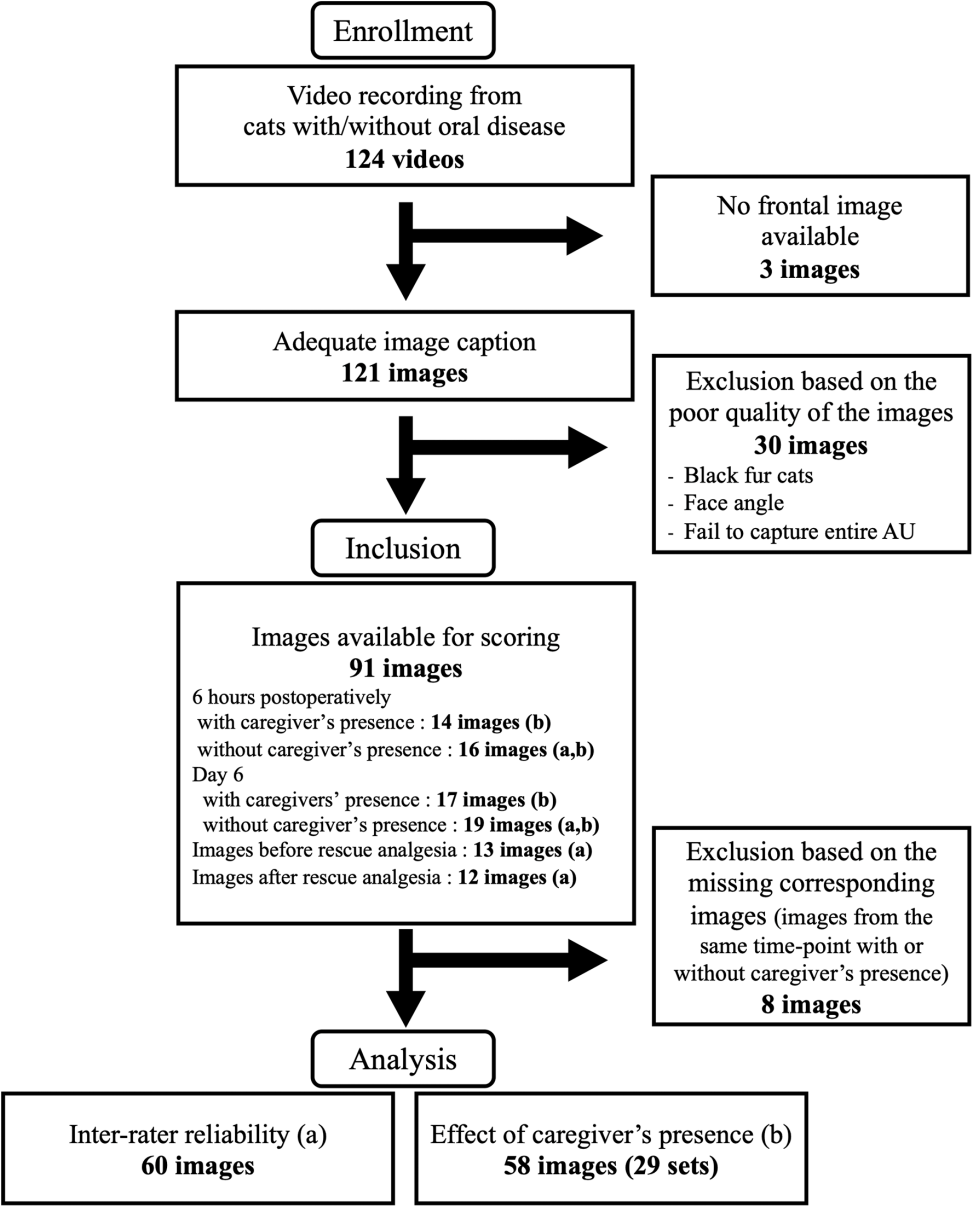
Flowchart of images captured from 24 cats with oral disease included in the study. Images with (a) and (b) were included for the analyses of inter-rater reliability and the effect of caregiver's presence, respectively.

Following video editing (trimming), a total of 124 videos were randomized using a random permutation generator (http://www.randomization.com) and renamed to consecutive numbers. Image capture (i.e., screenshots) of cat faces was performed for each video by a different investigator [GD] who was not involved with image scoring. Screenshots were performed when the cat was facing the camera and the entire face was visible. Then, the screenshot that was considered the most representative on the entire video for that timepoint was selected. Images were not captured if the cat did not face the camera at any time during the video (no frontal image). Quality assessment of each screenshot was performed by the same individual who edited the videos [RW]. Image quality was assessed based on the angle of the face, brightness, blur, and whether the entire face including ear tips, whiskers and part of the proximal scapula were visible (Figure 1).

### Image Scoring

A total of 91 images were independently scored by 4 raters [ME, BM, HR, PS, three Ph.D. candidates (female) and one board-certified veterinary anesthesiologist (male)] who were blinded to the oral conditions of cats and timing of the recording (Figure 1). The raters were supplied with the training manual published with the original article (6) (https://static-content.springer.com/esm/art%3A10.1038%2Fs41598-019-55693-8/MediaObjects/41598_2019_55693_MOESM1_ESM.pdf). Each image was evaluated using the FGS for scoring of five action units (AU): ears, eyes, muzzle, whiskers, and head position. The AUs were scored as following: 0 = AU is absent; 1 = moderate appearance of the AU, or uncertainty over its presence or absence; 2 = obvious appearance of the AU; or “not possible to score” = e.g., if the AU was not clearly visible (6). A total score was calculated by the sum of the scores of the AUs divided by the total possible score, excluding those marked as not possible to score (e.g., 3/8 = 0.375). The images were scored using an online survey (SurveyMonkey, https://www.surveymonkey.com) and divided into two sets. There was a minimum of 24 h and maximum of 48 h between scoring of the first and second set of images to avoid rater's fatigue. Scoring was performed between May 21st and 24th, 2019. Images receiving “not possible to score” for two or more AUs were excluded from statistical analyses.

### Statistical Analyses

Statistical analyses were performed using SPSS software (version 25.0 IBM SPSS Statistics, Armonk, NY, USA). Images from days 1 and 6 without the caregiver's presence and images before and after rescue analgesia were used for the analysis of inter-rater reliability. Images from days 1 and 6 with and without caregiver's presence were used for the analysis of effect of caregiver. Inter-rater reliability was calculated for each AU and for the total FGS score using intraclass correlation coefficients (ICC) with 2-way random effects ICC model for absolute agreement. ICC was interpreted according to a previously described scale (9): <0.5 = poor, 0.5–0.75 = moderate, 0.75–0.9 = good, and > 0.90 = excellent reliability. The ICC was calculated based on single measures (ICC_single_) which is an index for the reliability of the rating for one rater and the average of the measures (ICC_average_) which is an index for the reliability of mean of *k* raters as recommendation of the guideline (9). The effect of the caregiver's presence was assessed by comparing FGS scores of images with and without caregiver's presence using a Wilcoxon signed rank test. The FGS scores with and without the caregiver's presence were compared between no/minimal and severe oral disease cats using a Mann-Whitney *U*-test, and within each group using a Wilcoxon signed rank test. Normality of the distribution of the scores was assessed using a Shapiro-Wilk test. Values of *p* < 0.05 were considered statistically significant.

## Results

### Inter-rater Reliability

Sixty images without the caregiver's presence were included in the analysis. Images were available from days 1 and 6 (*n* = 16 and *n* = 19, respectively) and from before and after rescue analgesia from days 1, 2 and 3 (*n* = 13 and *n* =12, respectively) (Figure 1). Inter-rater reliability is presented in Table 1. ICC_single_ was moderate for ears, muzzle, whiskers, and head position and good for eyes. The ICC_average_ was good for muzzle and excellent for ears, eyes, whiskers and head position. Reliability of total FGS scores was good and excellent, based on ICC_single_ and ICC_average_, respectively.

**Table 1:** Inter-rater reliability of the Feline Grimace Scale in cats with oral disease.

**Action unit**		**ICC (95% CI)**
Ears	ICC_single_	0.68 (0.55–0.78)
	ICC_average_	0.89 (0.83–0.94)
Eyes	ICC_single_	0.76 (0.65–0.84)
	ICC_average_	0.93 (0.88–0.95)
Muzzle	ICC_single_	0.56 (0.43–0.69)
	ICC_average_	0.84 (0.75–0.90)
Whiskers	ICC_single_	0.64 (0.50–0.76)
	ICC_average_	0.88 (0.80–0.93)
Head position	ICC_single_	0.74 (0.63–0.82)
	ICC_average_	0.92 (0.87–0.95)
FGS total score	ICC_single_	0.84 (0.77–0.89)
	ICC_average_	0.95 (0.93–0.97)

*A total of 91 images were independently scored by 4 raters who were blinded to the oral conditions of cats and timing of the recording. Intraclass correlation coefficient (ICC) estimates with 95% confidence intervals (95% CI) were calculated based on single measures (ICC_single_) and average (ICC_average_) of measures, using a 2-way random effects model for absolute agreement. Interpretation of ICC was performed as following: ICC < 0.5 = poor, 0.5–0.75 = moderate, 0.75–0.9 = good, and > 0.90 = excellent reliability*.

### Effect of Caregiver's Presence

A total of 66 images were collected. From these, 29 images (13 and 16 sets from male and female cats, respectively) had a corresponding match (i.e., image from the same time-point with or without caregiver's presence), resulting in 58 images to be scored (day 1, *n* = 28 and day 6, *n* = 30). A total of 8 images did not have the corresponding match and were excluded (Figure 1). Median (range) of total FGS score without and with caregiver's presence were 0.088 (0–0.525) and 0.075 (0–0.325), respectively. Overall, there were not significant differences between scores with and without the caregiver's presence (*p* = 0.12). Median (range) of FGS scores without the caregiver's presence was 0.088 (0–0.325) in the minimal and 0.088 (0–0.525) in the severe group (*p* = 1.000). Median (range) FGS scores with the caregiver's presence in each group was 0.075 (0–0.325) in the minimal and 0.063 (0–0.250) in the severe group (*p* = 0.711). The FGS scores were not significantly different with or without the caregiver's presence within the no/minimal group (*p* = 0.195) or severe group (*p* = 0.398).

## Discussion

This study evaluated the inter-rater reliability of the FGS for pain assessment in cats with naturally occurring oral disease and the effect of the caregiver's presence on FGS scores. Overall, the results indicate that the reliability of each AU and total FGS scores based on ICC_single_ were moderate to good and that the presence of a male caregiver had no significant effect on the FGS scores.

Inter-rater reliability of total FGS scores was good to excellent considering ICC_single_ and ICC_average_. The estimate ICC_single_ is commonly used when a decision is made based on the scores of a single rater, however values of ICC_average_ are usually higher (9). In the current study, the inter-rater reliability for each AU was moderate (ears, muzzle, whiskers, and head position) to good (eyes). Reliability of scores of the muzzle and whiskers were lower than other AUs (ICC_single_ for muzzle and whiskers were 0.56 and 0.64, respectively). It is possible that dental extractions caused inflammation and facial edema likely impacting the scoring of muzzle and whiskers (i.e., difficulty of distinction between postoperative inflammation and the painful facial expression). Nevertheless, similar results were observed in the previous study in cats (0.63 and 0.55 for the muzzle and whiskers, respectively) (6). Reliability of the AUs ears and head position (0.68 and 0.74, respectively) were lower than the previous study (0.87 and 0.90, respectively) (6). In the present study, the camera was positioned to film the cats' behaviors for another study (3), and the height and angle of the video camera (set higher in the cage) may have not been ideal to capture the frontal image of the cat and further FGS scoring. If the camera angle is not optimal, the visualization, and interpretation of AUs could change between raters. However, ICC _single_ of total scores were good, and the result indicates that the raters could still identify the changes associated to pain in these cats.

In this study, 51.7% (15/29) of images with the caregiver's presence had lower, yet not significant, scores than those without caregiver's presence. Indeed, the caregiver's presence did not significantly affect the FGS scores either when data for each group were analyzed together or independently. On the other hand, a previous study reported that the presence of a male experimenter produced a stress-induced pain inhibition response in mice and rats (8). This previous study reported that this response disappears within 30–60 min and it is not known if longer acclimation periods would change the FGS scores with or without the presence of a caregiver in cats. Furthermore, in the present study, a male observer scored male and female cats during the study whereas male and female raters scored the images. The sex of the observer is known to affect real-time pain assessment in rodents (i.e., male pheromone induces analgesic effect) (8); similar findings have been reported with video-assessment in small animals (10). Although the present study was not specifically designed to evaluate the effect of sex on pain assessment, the presence of a male caregiver did not affect FGS scores via image assessment. However, it is not known if the sex of raters could have influenced FGS scores.

There are some limitations in this study. First, this was an exploratory study and the materials were obtained from previous reports (2, 3). As a result, sub-optimal image quality played an important role as discussed above. Indeed, 26.6% of the images were excluded. Additionally, power analysis and sample size calculation were not performed before the experiment because there is no consensus to determine the sample size a priori in the validation studies (11). Second, the order of video recording could not be randomized, and the videos without the caregiver's presence were always obtained before those with the caregiver's presence. However, this order bias was not present during image assessment because the videos were trimmed, and images were randomized before image selection and scoring by an observer not involved with image scoring. Third, images of cats presenting moderate (nine images) to severe (13 images) pain based on CMPS-F (3–4, and ≥ 5, respectively) were underrepresented. This could represent an important limitation to study the effects of caregiver's presence. If the images of painful cats were underrepresented, it is possible that some of these patients had low FGS scores which could not be significantly reduced during a stress-induced pain inhibition response with the caregiver's presence, as observed previously (8). One of the reasons for the lack of good quality images was that three black cats required rescue analgesia, and five of these images were excluded from analysis because identification of muzzle and whiskers were not possible in these individuals. This issue was also reported in previous studies in horses and cats (6, 12), and a possible solution would be the use of artificial lighting sources during recordings. The other possible way to balance the distribution of pain intensity across the images might be to obtain several screenshots from same painful time points (i.e., videos filmed before rescue analgesia). However, the increase of number of images from same cats could bias the raters' scores. Finally, images of days 1 and 6 were included for the analysis of the effect of caregiver's presence. The images obtained on day 6 might have biased the results since perhaps cats were no longer painful. However, the pain scores (CMPS-F) in the severe group were significantly higher than the minimal group on day 6 (2), which made the authors believe cats in severe group could still be in mild pain.

In conclusion, the FGS is a reliable tool for assessment of oral pain in cats, though some action units were difficult to identify due to poor image quality and facial edema and inflammation. The caregiver's presence did not affect the FGS scores. The influence of sex in the FGS scores should be a subject of future investigations.

## Data Availability Statement

The datasets generated for this study are available on request to the corresponding author.

## Ethics Statement

The animal study was reviewed and approved by Comité d'éthique de l'utilisation des animaux (CÉUA); Faculté de médecine vétérinaire, Université de Montréal. Written informed consent was obtained from the owners for the participation of their animals in this study.

## Author Contributions

RW and PS designed, conducted the study, and drafted the manuscript. RW performed postoperative care and pain assessment, the video filming, the image selection and the statistical analyses. GD performed the general anesthesia and the image captures. ME, HR, BM, and PS scored the images. All authors reviewed and approved the final manuscript.

## Conflict of Interest

The authors declare that the research was conducted in the absence of any commercial or financial relationships that could be construed as a potential conflict of interest.
